# Disparities in heart transplant survival and graft rejection outcomes persist in the modern era: a call to race towards a more equitable future

**DOI:** 10.1093/ejcts/ezaf141

**Published:** 2025-04-16

**Authors:** Ahad Firoz, Daniel Remer, Huaqing Zhao, Xiaoning Lu, Eman Hamad

**Affiliations:** Department of Internal Medicine, University of California Davis Medical Center, Sacramento, CA, USA; Center for Urban Bioethics, Lewis Katz School of Medicine, Philadelphia, PA, USA; Department of Biomedical Education and Data Science, Lewis Katz School of Medicine, Philadelphia, PA, USA; Department of Biomedical Education and Data Science, Lewis Katz School of Medicine, Philadelphia, PA, USA; Department of Medicine, Section of Cardiology, Temple University Hospital, Philadelphia, PA, USA

**Keywords:** heart transplantation, disparities, race, survival, graft rejection, UNOS

## Abstract

**OBJECTIVES:**

Racial and ethnic disparities remain an ongoing challenge in healthcare. Such inequities have been reported in heart transplantation (HTx); however, there is limited data within the modern era. Additionally, there is scarce information on outcomes besides death, such as graft rejection. Therefore, our investigation aims to add further data on contemporary racial and ethnic disparities on post-transplant outcomes.

**METHODS:**

Adult isolated HTx recipients who were transplanted between 1/2000 and 9/2023 were analysed using the United Network for Organ Sharing (UNOS) database. Inclusion criteria included ‘White’, ‘Black’, ‘Hispanic’ and ‘Asian’ recipients. Two primary outcomes of interest were analysed: mortality and cardiac allograft vasculopathy (CAV). Survival was assessed using a cause-specific model, whereas CAV analysis utilized a competing-risk approach. Subgroup survival analysis was conducted for patients listed in the years prior to (11/2013–10/2018) and after (10/2018–9/2023) the 2018 heart allocation policy (HAP) changes.

**RESULTS:**

A total of 50 243 patients were included in our analysis. Black recipients were the only group found to have an increased overall (hazard ratio [HR] = 1.30, *P* < 0.001) and post-HAP (HR = 1.36, *P* < 0.001) mortality risk. Asian (HR = 1.19, *P*= 0.001) and Hispanic (HR = 1.15, *P* < 0.001) recipients had elevated risks of CAV, whereas Black patients had similar risk (HR = 1.00, *P* = 0.864) as White recipients.

**CONCLUSIONS:**

Our investigation suggests that disparities continue to exist for minority groups after HTx. Notably, the 2018 allocation changes may have introduced or exacerbated such inequities for Black recipients.

## Graphical Abstract

**Figure:**
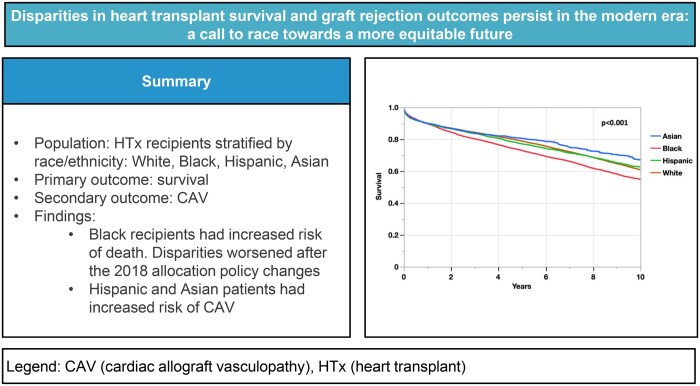

## INTRODUCTION

Despite exponential advancements in medicine and technology, our modern society continues to grapple with long-standing social issues, such as racial inequality. The concept of race, a pseudoscientific social construct, was originally created many centuries ago as a means to justify slavery, colonization and imperialism [1]. Even today, the power differential between White and minority races still exists and is particularly noticeable within the US healthcare system. With little genetic basis, the inequities observed within this model are largely the result of structural racism and discriminatory practices that target marginalized groups across multiple metrics, leading to difficulties in accessing medical care and even contributing to suboptimal outcomes [2]. Such disparities are especially evident in the nation’s leading cause of morbidity and mortality, heart disease [3].

In the field of cardiovascular medicine, though exceptional screening and medical breakthroughs have taken place, the benefits of such innovations are not equally shared across all groups [4]; this remains true even for advanced therapies, such as ventricular assist devices (VADs) and heart transplantation (HTx) [5]. Within HTx, differences in outcomes have been reported both on the waitlist and after transplantation for minority groups [6, 7]. For instance, in a retrospective investigation that utilized data from the United Network for Organ Sharing (UNOS) between 1987 and 2009, the authors found that Black recipients had a 34% increased long-term mortality risk compared to White groups, even after accounting for demographic and socioeconomic factors [6]. Though such disparities may not be unique to transplant medicine, for the field of HTx in particular, a gold-standard, life-saving intervention with limited resources, extra precautions must be taken to ensure both beneficence and justice for all patients who are in need of a second chance at life. As such, continued research is necessary to identify and address such inequities urgently. Currently, some of the literature on this topic is outdated, with occasional reports utilizing cases since the inception of HTx in the USA during the 1980s; while this data is still relevant for our understanding of racial and ethnic disparities in HTx and its progress over the years, it may nonetheless skew the social inequities that certain groups face in the present day. Additionally, there is a paucity of studies which incorporate updated data from the current era, and even fewer that examine the impact of the 2018 heart allocation policy (HAP) changes on minority group disparities. Lastly, the role of race and ethnicity on other post-transplant outcomes besides survival, such as graft rejection, has been rarely studied. Therefore, our investigation sought to identify and address contemporary disparities in outcomes that minority groups may face after HTx.

## METHODOLOGY

### Data collection and study cohort

We conducted a retrospective cohort analysis using the Organ Procurement and Transplantation Network (OPTN) thoracic database on adult (≥18 years) orthotopic HTx patients transplanted between January 2000 to September 2023. Patients with a previous history of HTx, or recipients who ultimately received a heterotopic HTx or multi-organ transplant were excluded. Only patients with a racial or ethnic demographic of ‘White’, ‘Black’, ‘Hispanic’ and ‘Asian’ were included in the final cohort. The UNOS database recorded these race and ethnicity groups as mutually exclusive to each other.

### Data analysis

Statistical analyses comparing racial and ethnic groups were conducted in order to better describe the study sample. Categorical variables were listed as a percentage of the sample cohort and were compared using Pearson chi-square tests. Continuous variables were either given as a mean ± standard deviation if normally distributed, or as a median (25th–75th percentile) if non-normally distributed; analysis of variance (ANOVA) or non-parametric testing was conducted between groups, respectively.

Post-HTx survival and graft rejection in the form of cardiac allograft vasculopathy (CAV) was analysed using time-to-event modelling. Mortality was analysed using a cause-specific approach, including Kaplan–Meier curves, log-rank tests and Cox regression models. In addition to analysing the entire sample cohort, further subgroup analyses were conducted to identify the role of the 2018 HAP changes on racial and ethnic disparities. This was done by comparing subacute survival for patients who were listed for HTx immediately before (pre-HAP era: 1 November 2013–17 October 2018) and after (post-HAP era: 18 October 2018–30 September 2023) the modifications were implemented. In addition to mortality, CAV development was studied utilizing a competing risk approach [8], with death as the competing event; this included cumulative incidence plots, Gray’s tests and Fine-Gray subdistribution models. For this investigation, CAV was defined as a diagnosis of coronary artery disease (CAD) at the time of follow-up. A detailed description of the multivariable model construction and list of potential covariables is included in the Supplementary Materials.

As analyses were exploratory in nature, a *P*-value less than 0.05 was considered statistically significant throughout the entirety of the study. All analyses were performed using JMP Pro 16 or SAS 9.4 software (SAS Institute Inc., Carry, NC).

## RESULTS

### Participants and transplantation trends

**Figure 1:**
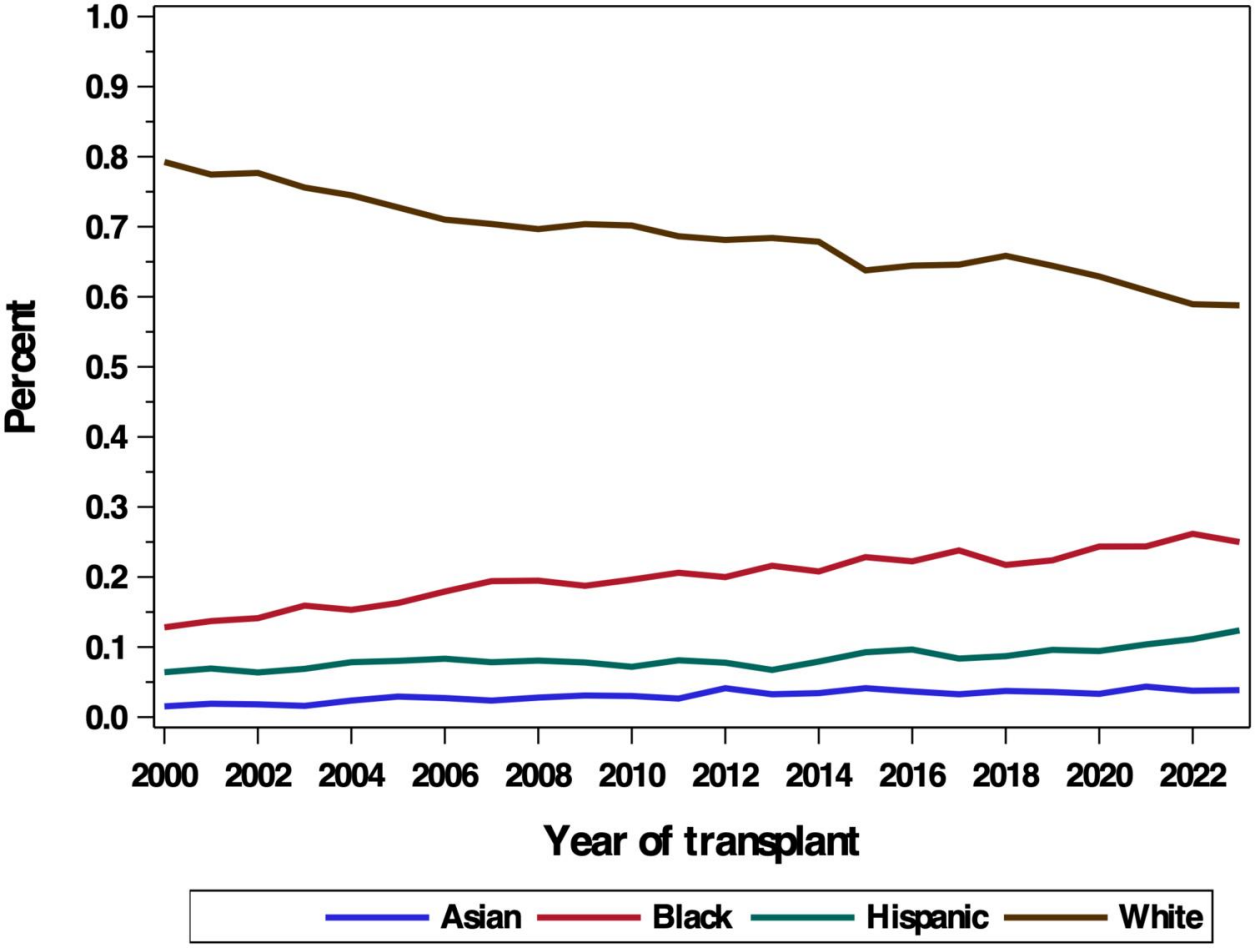
Yearly trends in heart transplantation. Approximate yearly rate of change with Cochran–Armitage testing: White: −0.79% (ref.); Black: +0.52% (*P* < 0.001); Hispanic: +0.18% (*P* < 0.001); Asian: +0.10% (*P* < 0.001).

In total, 50 243 recipients were included in the analysis, of which 67.6% were White, 20.6% Black, 8.6% Hispanic and 3.2% Asian. The yearly heart transplant trends by group are shown in Fig. 1. The proportion of White patients transplanted decreased over time, while Black and Hispanic groups increased gradually. The frequency of Asian patients transplanted remained approximately constant. Cochran–Armitage testing found a significant difference in yearly trends between White patients and all minority groups (*P* < 0.001).

### Descriptive data

Demographic data for each group are presented in Supplementary Material, Table S1. White patients were typically older and had the highest proportion of cigarette users and a heart failure aetiology of ischaemic cardiomyopathy (ICM). They also had the longest stay on the waitlist until transplantation. Black candidates were typically younger and had the greatest frequency of females, nonischemic dilated cardiomyopathy (NIDCM), inotrope use, VAD or total artificial heart (TAH) and high-acuity status at the time of transplant. Conversely, they also had the lowest proportion of patients who were low acuity at transplant. The Hispanic group had the highest proportion of recipients with an educational level of high school or below and Medicaid or other need-based coverage. Asian recipients generally had the lowest weights and BMI, an educational level of college or above with private or self-funded insurance, shortest length of time on the waitlist and the highest incidence of transplantation at high-volume centres (>35 cases per year, on average).

Donor and donor–recipient matching information for each group is listed in Supplementary Material, Table S2. White recipients had the greatest proportion of donors who were cigarette users. Similar to recipient demographics, Black patients had the highest incidence of female donors. The highest frequency of each donor race and ethnicity belonged to the same corresponding recipient group. Ischaemic time was similar across all groups. Asian recipients were generally matched with lower weight donors and had the highest donor-to-recipient predicted heart mass ratio. Black patients generally had the most total HLA and HLA-A mismatch with their donors, while Asian recipients had the greatest frequency of mismatch at the HLA-B and -DR loci.

Transplant peri- and postoperative data are displayed in Supplementary Material, Table S3. Patients who were White were most likely to not require hospitalization prior to transplantation. In contrast, those who were Asian had the greatest proportion of recipients waiting in the intensive care unit (ICU). At the time of transplant, Black recipients had the highest frequency of IL-2 receptor antagonist use as an induction agent, while Asian patients had the lowest. The opposite relationship was seen for polyclonal ALG/ATG induction agents. Steroid induction was greatest in Black and Hispanic groups. Following transplantation, though statistically significant, length of stay (LOS) between groups was clinically similar. Prior to discharge, Black recipients had the greatest incidence of acute kidney injury necessitating dialysis, whereas White patients had the highest frequency of permanent pacemaker placement. There was no significant difference in the incidence of stroke prior to discharge between groups (*P* = 0.194). Black recipients had the highest prevalence of acute rejection prior to discharge and within 1-year of transplant, whereas Asian patients had the lowest frequency. Out of the recipients who died, Black recipients had the highest incidence of cardiovascular and graft failure causes of death.

### Survival outcome and HAP analysis

**Figure 2:**
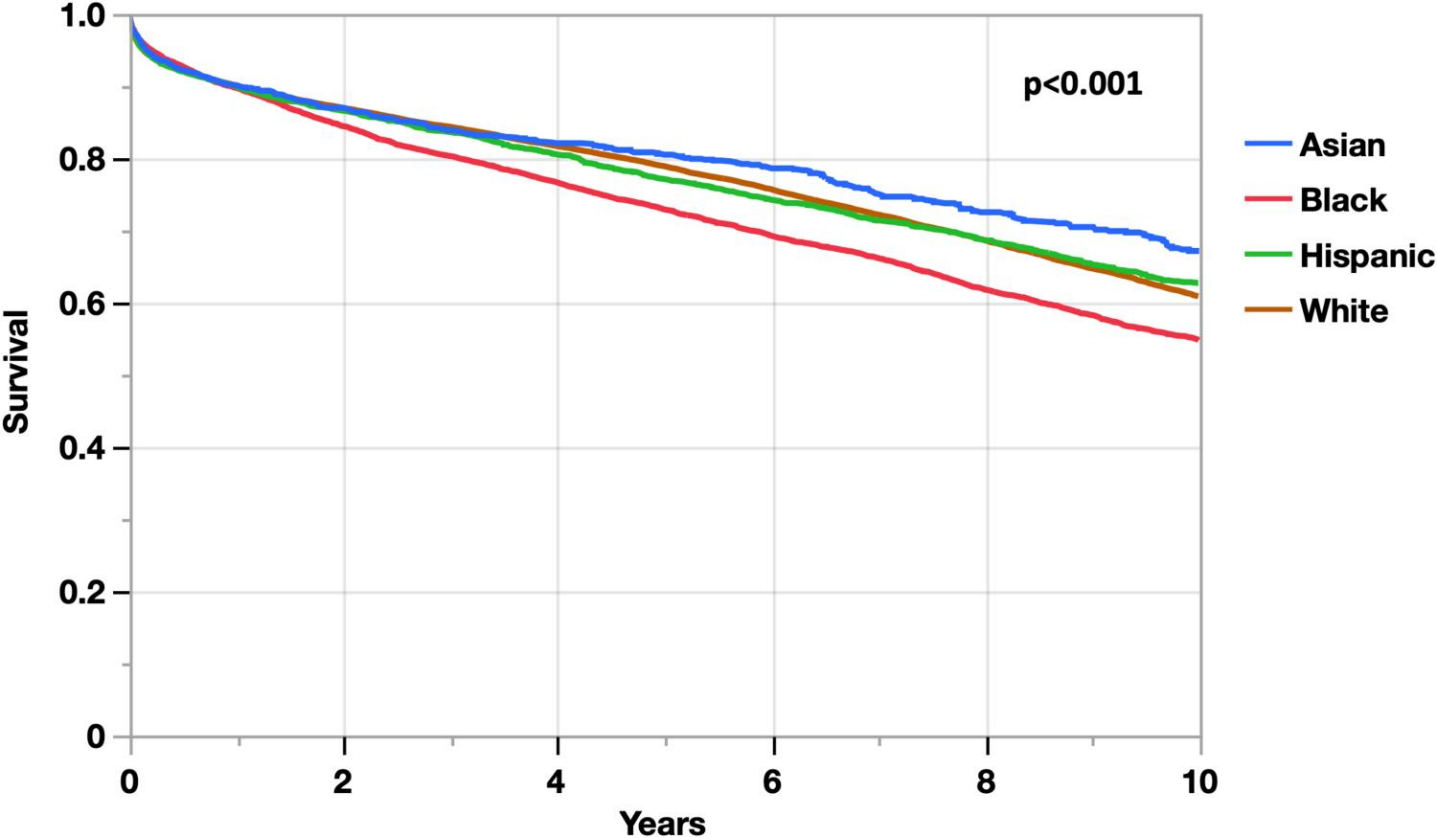
Kaplan–Meier survival curves following heart transplantation.

Kaplan–Meier survival curves stratified by recipient race and ethnicity are illustrated in Fig. 2. Log-rank test found a significant difference in mortality between groups (*P* < 0.001). Multivariable Cox regression model was created for the transplanted cohort; results are displayed in Table 1. Out of the four categories, Black recipients were the only group with a significant difference in mortality risk compared to White patients (hazard ratio [HR] = 1.30, *P* < 0.001). Additional subgroup survival analysis on HAP era was conducted, for which the Cox regression model results are also included in Table 1. Race and ethnicity did not play a significant role on subacute mortality during the pre-HAP era; however, in the post-HAP era, Black race was again found to be the only group with an elevated mortality risk with reference to White recipients (HR = 1.36, *P* < 0.001).

**Table 1:** Cox regression survival models

	Unadjusted model		Final model	
	HR (95% CI)	*P*-value	HR (95% CI)	*P*-value
Overall^a^				
White	Ref.	–	Ref.	–
Black	1.23 (1.18–1.28)	<0.001	1.30 (1.25–1.36)	<0.001
Hispanic	0.99 (0.93–1.05)	0.770	0.98 (0.92–1.05)	0.566
Asian	0.86 (0.78–0.96)	0.007	0.90 (0.80–1.00)	0.058
pre-HAP era (1 Nov. 2013–17 Oct. 2018)^b^				
White	Ref.	–	Ref.	–
Black	1.09 (0.97–1.23)	0.146	1.11 (0.98–1.25)	0.106
Hispanic	1.14 (0.96–1.35)	0.133	1.11 (0.94–1.32)	0.229
Asian	1.14 (0.89–1.48)	0.298	1.11 (0.86–1.44)	0.430
post-HAP era (18 Oct. 2018–30 Sept. 2023)^b^				
White	Ref.	–	Ref.	–
Black	1.24 (1.10–1.40)	<0.001	1.36 (1.20–1.55)	<0.001
Hispanic	0.98 (0.81–1.17)	0.795	1.01 (0.83–1.22)	0.938
Asian	0.98 (0.74–1.31)	0.901	1.04 (0.78–1.39)	0.791

a Survival during a 10-year period.

b Survival during a 3-year period.

HAP: heart allocation policy; HR: hazard ratio; Ref.: reference group.

To better understand what factors may be driving the recently observed disparity for Black recipients compared to White groups, a detailed analysis comparing demographic factors between these two races in the pre- and post-HAP era was conducted, as included in Supplementary Material, Table S4. Between the eras, the incidence of diabetes, an educational level of high school or below, insurance of Medicaid or donation payment and high-acuity status in Black groups increased disproportionately relative to White recipients. Donor and donor–recipient matching data remained clinically similar.

### CAV outcome

**Figure 3:**
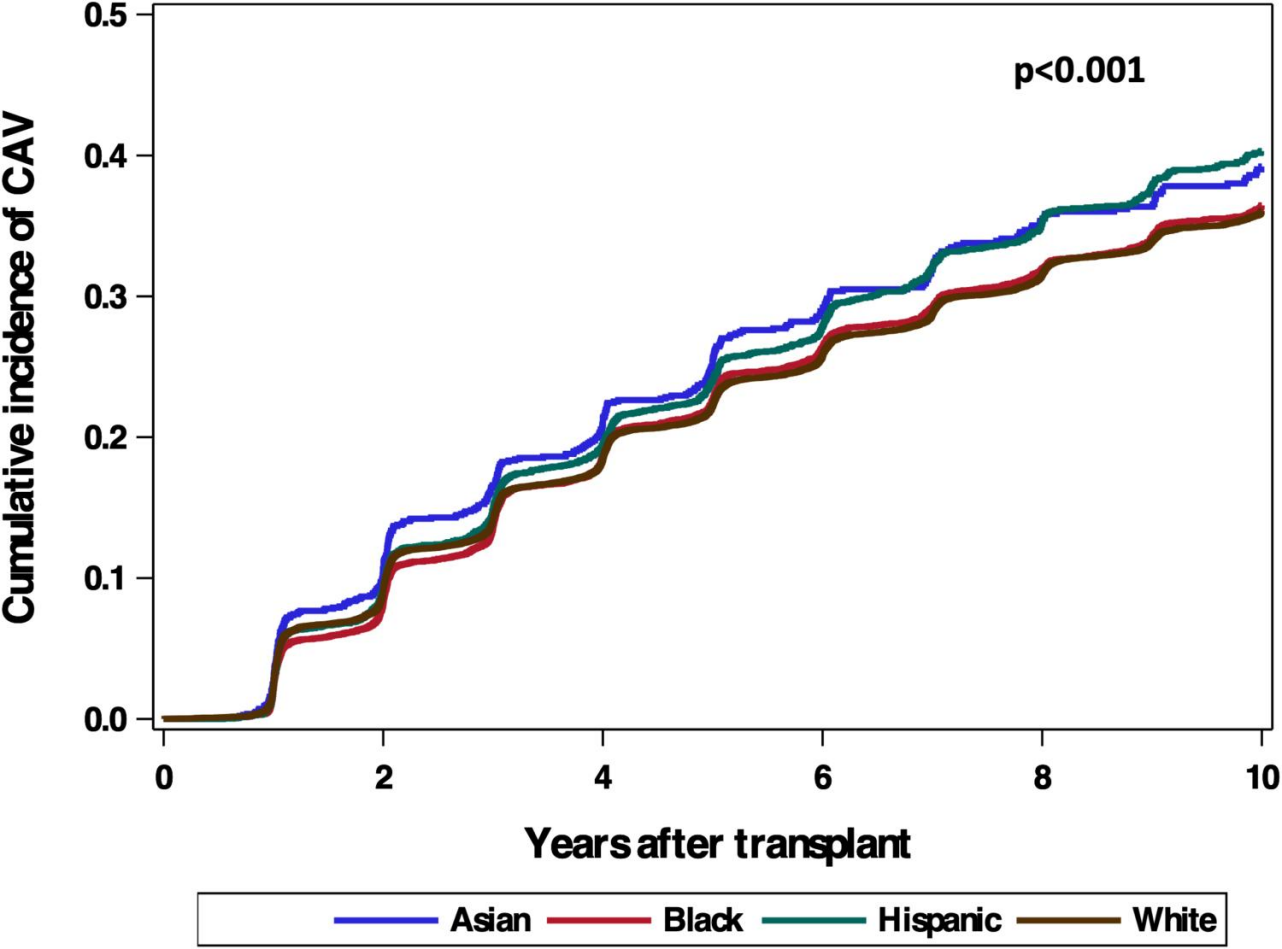
Cumulative incidence function for CAV development. CAV: cardiac allograft vasculopathy.

In addition to mortality, CAV outcomes were also analysed between race groups. Cumulative incidence function of CAV for each group is displayed in Fig. 3. Gray’s test found a significant difference in time-to-CAV between groups (*P* < 0.001). Multivariable Fine-Gray subdistribution model in Table 2 found that Hispanic (HR = 1.15, *P* < 0.001) and Asian (HR = 1.19, *P* = 0.001) recipients had an elevated risk of CAV, whereas Black patients (HR = 1.00, *P* = 0.864) had a similar risk as White recipients.

**Table 2:** Competing risk models for CAV development

	Unadjusted model		Final model	
	HR (95% CI)	*P*-value	HR (95% CI)	*P*-value
CAV development^a^				
White	Ref.	–	Ref.	–
Black	0.99 (0.95–1.03)	0.640	1.00 (0.96–1.05)	0.864
Hispanic	1.11 (1.04–1.18)	0.001	1.15 (1.08–1.23)	<0.001
Asian	1.11 (1.01–1.23)	0.039	1.19 (1.07–1.32)	0.001

a CAV development during a 10-year period.

CAV: cardiac allograft vasculopathy; HAP: heart allocation policy; HR: hazard ratio; Ref.: reference group.

## DISCUSSION

To date, this investigation is among the largest retrospective studies that examine racial and ethnic disparities on modern heart transplant outcomes. Though transplantation rates have increased steadily for Black and Hispanic patients, clear and persistent disparities continue to exist for marginalized groups. Our analysis found that Black recipients had higher rates of dialysis and acute rejection prior to discharge, treatment for acute rejection within 1-year of transplant and a 30% increased long-term mortality risk compared to those who were White. This finding is relatively well-established in the current literature for post-transplant mortality [6, 9, 10].

As with many processes, the mortality disparity identified in this study is likely heavily impacted by barriers to healthcare and social determinants of health (SDOH). For instance, in a study conducted by Kilic *et al.* [9], the authors found that Black patients were more likely to be transplanted at suboptimal performing centres. Another component to consider is the hefty cost of care after transplantation. Our report found that Hispanic and Black patients had the highest rates of pro-bono or government sponsored payer systems for HTx. Depending on their coverage, such patients may have greater financial difficulties in affording specialist follow-up appointments, acute hospitalizations and immunosuppressive drug regimens compared to those with private insurance, thereby potentially leading to non-adherence and worse outcomes [11]. Other SDOH include the physical environment of recipients; in this regard, Wayda *et al.* [10] investigated the role of neighbourhood socioeconomic status (SES) through patient zip code data and found Black recipients to be the only racial group with increased post-HTx mortality. Yet, despite the inclusion of multiple variables that may act as a proxy for healthcare inequities and SDOH, it is nonetheless difficult to factor into computational models the generations of discriminatory practices, disenfranchisement and structural racism that affect people of colour and minority groups. For instance, within the subspecialized field of advanced heart failure and transplantation, there are reports which demonstrate implicit biases from providers, particularly older professionals, that may favour White over Black candidates for HTx listing [12]. Such biases and delays in HTx referral may in part explain why our investigation found Black recipients to have a higher acuity status, likely illustrating advanced disease. This is significant as longer wait times for a new heart has been associated with increased risk of post-HTx graft failure and death [13].

In addition to differences in social status, our investigation found significant clinical variations between groups. For instance, patients who were Black had the highest incidence of NIDCM as their primary heart failure aetiology, compared to White recipients who had the greatest proportion of ICM. Although prior studies have found patients with NIDCM to have superior survival outcomes compared to those with ICM [14], it remains unclear why these findings do not translate across races. Additionally, Black recipients were more likely to be bridged with VAD/TAH compared to other groups. A similar finding was noted by Breathett *et al.* [12] in their qualitative study that surveyed and interviewed heart failure professionals; in this investigation, the authors observed that participants were more likely to offer White groups transplantation and Black patients VAD, again highlighting inequities in advanced heart failure allocation. While left ventricular assist devices as a bridge to transplantation have prolonged survival for eligible patients, their use has nonetheless been associated as a poor prognostic factor when compared to those with a de-nova HTx [15]. Conversely, as race is a social construct, biologic and genetic factors likely have less of a role in outcomes compared to SDOH; however, a commonly cited immunogenetic determinant is HLA mismatching and their antibody formation. While our study did find that Black recipients had the greatest level of total HLA mismatches with their donors, current research suggest that HLA mismatching at the conventional -A, -B and -DR loci may not significantly impact long-term survival in today’s era, thereby partially negating the previous claim on the role of HLA matching in HTx disparities [16].

Additionally, our investigation discovered that Hispanic and Asian patients had comparable survival as the reference group. Though other studies found similar results with respect to Hispanic patients, there is scarce data on outcomes in Asian recipients [9, 10]. Notably, our investigation found Asian recipients to have the best survival based on Kaplan–Meier survival curve estimates; such patients even had a survival advantage in a univariable Cox regression model. Interestingly, this group also had the highest measures for variables that acted as proxies for SES, such as educational level and insurance. However, after accounting for such SDOH in the fully adjusted model, this group was found to have no significant difference in mortality risk compared to White patients. This finding further establishes the concept that race is merely a social construct, with limited-to-no inherent biologic or causative role on patient outcomes.

A major change that occurred during the study period was the introduction of a new allocation system that aimed to reduce waitlist mortality and prioritize transplantation for high acuity patients. In brief, prominent changes that were introduced in the 2018 HAP include the transition from a 3- to 6-tiered allocation system to better characterize patient acuity level and also serve those with rarer forms of cardiomyopathy, as well as the extension of donation service areas to encompass larger geographic zones [17]. In investigating the impact of such changes, the period just prior to these modifications was analysed as a control; during this time, we found that there was no significant difference in subacute survival between groups, supporting the notion that disparities had been somewhat improving. An investigation that studied the interaction between time period and disparities proposed a similar conclusion [18], though there remains conflicting data in the literature [19]. In analysing recipients transplanted in the post-HAP era, our analysis found that Black patients had an increased mortality risk compared to those who were White. While there is ongoing research to better understand the overall impact and implications of the 2018 HAP changes, there are limited studies which have analysed its role through the lens of racial and ethnic disparities. However, in one recent study conducted on this topic, Sherard *et al.* [20] found no significant difference in survival outcome between racial groups following the new allocation changes during a study period of October 2018 to September 2021. Besides differences in cohorts, one apparent explanation for the contrasting results is the referenced study duration constituting less than 3-years, which likely limits the analysis for most patients to a comparatively shorter, more acute follow-up period.

Though the recent increase in mortality risk for Black patients relative to White groups is most likely multifactorial in origin, the Covid-19 pandemic may have contributed to such findings. In the literature, it is already well reported that the SARS-CoV-2 virus has disproportionately affected communities of colour through increased infection, hospitalization, and mortality; however, Black groups have also received a greater share of the socioeconomic burden of the pandemic, including increased unemployment rates and food insecurities [21]. The socioeconomic impact of the pandemic is evident from our investigation when the pre- and post-HAP eras were compared, with increased disparities noted for Black recipients in their education and health insurance demographics. Collectively, these increased social stressors may introduce new or further exacerbate existing barriers to healthcare in the Black community, thus resulting in suboptimal outcomes compared to other groups. In addition to the widened disparities attributed to the pandemic, another causative factor to consider is the intrinsic system of the new HAP changes, namely its primary purpose to reduce waitlist mortality. While studies have found that the HAP modifications have been successful in meeting its goal, a direct consequence of this policy is the increased transplantation of high acuity patients, which in turn likely played a major role in the observed diminished post-transplant outcomes compared to prior years [22]. In this regard, our analysis discovered that the recruitment of high allocation status patients were not uniform across groups; namely, Black recipients had a disproportionate rise in acuity status compared to White groups in the post-HAP era. Although this may represent a positive change, namely the increased recruitment of higher-risk Black patients who would have previously not received a HTx, this asymmetry in clinical status between White and Black may nonetheless contribute in part towards their observed survival difference.

After recognizing disparities for both recent and overall mortality within the Black community, it becomes necessary to identify solutions to address such inequalities. First, given the inseparable link between racial disparities and SDOH, it is imperative to enact social policies that serve to eliminate barriers to healthcare; such policies may be targeted towards reinvesting in low income communities or neighbourhoods that were traditionally redlined to ensure safe and affordable housing, improve accessibility to healthy foods, offer greater transportation opportunities, increase educational funds for underserved public schools and provide sufficient healthcare coverage [23]. Additionally, increased effort should be made to diversify the field of cardiovascular medicine, and medicine as a whole, with groups that are traditionally under-represented (URM); increased URM representation has been shown to improve marginalized community’s trust in the healthcare system, adherence to standard of care therapy, recruitment in clinical trials and overall medical outcomes [24]. Similarly, at the provider level, regular screening and training opportunities may be provided for implicit bias recognition and self-awareness, though there is still ongoing research on its impact [25]. Lastly, we encourage transplant databases to collect and include more granular information on patient social history; only by understanding the root cause may we improve the disparities that marginalized communities face.

In addition to mortality assessment, our investigation also analysed CAV outcomes. Limited data currently exist on the role of race or ethnicity in chronic graft rejection, particularly for HTx. Interestingly, we found that Hispanic and Asian groups had increased risk of developing CAV compared to White recipients, whereas Black patients had an overall similar outcome. Though there are scarce data in the literature on the topic, some studies report that Black recipients are at an increased risk for CAV [19, 26]. However, one significant limitation to these investigations is their use of cause-specific, Cox regression modelling which traditionally censors death; though the results remain useful, this approach for time-to-disease occurrence may represent a violation of the independent and noninformative censoring assumption of the model [27]. To the best of our knowledge, our investigation is the first to describe the role of race and ethnicity on HTx chronic graft rejection outcomes through a competing risk model. This approach is especially important given the established mortality risk in the Black HTx recipient population, and the association between CAV and death [28].

The currently known risk factors for CAV are generally grouped into immune and non-immune mediated aspects [29]. Immunologic risk factors include HLA mismatching with the subsequent development of anti-HLA antibodies or other donor-specific antibodies (DSA). Of the 3 loci, -A, -B and -DR, mismatches at the HLA-DR loci were found to be most predictive of CAV development [16], of which Asian patients had the greatest level of complete mismatch. Non-immunologic predictors of CAV include risk factors for CAD, for both the recipient and donor. Using ICM aetiology as an approximate proxy for recipient CAD risk factors, Black patients had the lowest risk while Asian candidates had over double the proportion of ICM. Still, an important consideration given the disparities in medical care may be that CAV is under-diagnosed for Black patients. Though not specific to HTx recipients, a retrospective analysis conducted by Desai *et al.* [30] found that among patients who were hospitalized for multivessel percutaneous intervention, Black patients had significantly lower utilization of coronary angiography, left heart catheterization and intravascular ultrasound. The potential for under-diagnosis of CAV in Black recipients is paradoxically further supported in our study by this group’s elevated incidence of cardiovascular and graft failure causes of death, suggesting that there may be a hidden component of CAV for many of these deceased patients that was not diagnosed. Additionally, because Black patients traditionally have been observed to have greater immunologic risk factors, namely increased de-novo DSA (dnDSA) formation and acute graft rejection episodes and severity [31, 32], their theoretical risk of CAV should be increased relative to White patients.

### Limitations

Our analysis is limited by several factors common to all studies that utilize a national database, such as missing or incorrectly entered data. Similarly, limited data on patient social history may result in absent confounding variables which may better explain the racial and ethnic disparities noted throughout the investigation. In other circumstances, there may exist no ideal measurement for a certain variable; for example, describing the generations of systemic racism, prejudice and biases that certain groups face. Additionally, many measures of SES are not fixed values and may change during the course of the listing or post-transplantation process, which can result in data points that are not reflective of the true SES or experienced SDOH of patients. Lastly, although the collection of recipient information and outcomes from centres across the USA may increase the generalizability of the study, it comes at the expense of reduced internal validity. This is especially applicable to the recorded data on CAV, which lacks granularity on methodology and testing indication, diagnostic criteria and disease severity among other variables. Further investigation is warranted using a standardized protocol for the testing and diagnosing of CAV to better understand the true role of race and ethnicity on CAV development.

## CONCLUSION

Our analysis found evident disparities in post-heart transplant outcomes during the 21st century for minority groups. Black recipients were found to have an increased overall long-term mortality risk compared to White patients. These disparities worsened for Black groups following the 2018 HAP changes, suggesting such modifications may have introduced or exacerbated inequalities. Additionally, while Hispanic and Asian recipients had comparable overall survival as the reference group, they were discovered to have an increased risk of CAV. These results necessitate a call to action: further work must be done on a provider, institutional and societal level to address barriers to healthcare and ultimately eliminate disparities for a more equitable future.

## Supplementary Material

ezaf141_Supplementary_Data

## Data Availability

Requests to access the data underlying this article should be directed to the United Network for Organ Sharing.

## ABBREVIATIONS

CAD: Coronary artery disease
CAV: Cardiac allograft vasculopathy
DSA: Donor-specific antibodies
HAP: Heart allocation policy
HTx: Heart transplantation
HR: Hazard ratio
ICM: Ischaemic cardiomyopathy
NIDCM: Nonischemic dilated cardiomyopathy
SDOH: Social determinants of health
SES: Socioeconomic status
UNOS: United Network for Organ Sharing
VAD: Ventricular assist device
