# Supplementary material for: Disparities in heart transplant survival and graft rejection outcomes persist in the modern era: a call to race towards a more equitable future
Source: Eur J Cardiothorac Surg. 2025 Apr 16;67(4):ezaf141. doi: 10.1093/ejcts/ezaf141 (PMC12036964; doi:10.1093/ejcts/ezaf141)
Supplement: ezaf141_Supplementary_Data [file ezaf141_supplementary_data.docx]

*Multivariable model construction*

Multivariable models for both survival and CAV analyses were created using the following clinically relevant covariables: recipient age, sex, race and ethnicity, weight, history of diabetes and cigarette use, heart failure etiology, transplant period, highest educational level, insurance type, ECMO, IABP, inotrope, VAD/TAH, and ventilator use at the time of registration, end acuity status, days on waitlist, annual HTx center volume, donor age, sex, race and ethnicity, weight, history of hypertension, cigarette use (>20 pack-years), and diabetes, cause of death, ischemic time, HLA mismatch, IL-2, polyclonal ALG/ATG, and steroid induction use. For survival models, total HLA mismatch was utilized, whereas for CAV models, HLA-A, -B, and -DR loci mismatch were used. All covariables with a p-value less than 0.10 in a univariable model were entered into the multivariable model. The final adjusted model was created using a backward stepwise elimination method until all covariables had a p-value of less than 0.05.

*Data statement*

The data underlying this article was provided by UNOS. Requests to access this data should be directed to UNOS.

**Supplementary Table S1:** Recipient demographics

|  | White  (n=33,977) | Black  (n=10,345) | Hispanic  (n=4,331) | Asian  (n=1,590) | *p-value* |
| --- | --- | --- | --- | --- | --- |
| Age (years) | 57 (49-63) | 52 (41-60) | 53 (42-60) | 54 (44-62) | <0.001 |
| Sex (male) | 76.6% | 66.8% | 74.5% | 76.9% | <0.001 |
| Weight (kg) | 83.5±17.2 | 84.6±18.9 | 77.1±16.6 | 68.3±14.9 | <0.001 |
| BMI (kg/m^2^) | 27.3±4.7 | 27.7±5.2 | 26.9±4.8 | 24.2±4.3 | <0.001 |
| Primary etiology  NIDCM  ICM  HCM  RCM | 44.4%  42.5%  3.0%  3.2% | 74.5%  17.6%  1.1%  3.3% | 57.7%  31.7%  2.2%  2.1% | 48.5%  38.7%  3.7%  2.5% | <0.001 |
| History of diabetes | 25.1% | 27.2% | 31.5% | 31.3% | <0.001 |
| History of cigarette use | 48.7% | 41.0% | 38.7% | 35.4% | <0.001 |
| Highest educational level  High school or below  College or above | 41.0%  59.0% | 49.3%  50.7% | 64.5%  35.5% | 28.3%  71.7% | <0.001 |
| Insurance type  Medicaid or donation  Medicare or other govt. sponsored  Private or self-funded | 19.3%  21.7%  59.0% | 32.6%  23.7%  43.7% | 36.8%  22.1%  41.1% | 24.3%  14.0%  61.8% | <0.001 |
| ECMO* | 1.4% | 1.2% | 1.4% | 2.6% | 0.001 |
| IABP* | 7.3% | 9.5% | 8.9% | 10.3% | <0.001 |
| Inotrope use* | 32.3% | 41.0% | 40.5% | 38.1% | <0.001 |
| VAD/TAH* | 22.4% | 27.6% | 21.8% | 23.2% | <0.001 |
| Ventilator* | 2.2% | 1.5% | 1.9% | 2.6% | <0.001 |
| Days on WL | 77 (20-248) | 67 (19-228) | 54 (15-201) | 35 (11-134) | <0.001 |
| Acuity status at transplant  Low^1^  Medium^2^  High^3^ | 11.7%  31.4%  56.9% | 5.8%  29.3%  64.9% | 9.8%  28.1%  62.1% | 9.3%  27.8%  63.0% | <0.001 |
| Transplant period  2000-2007  2008-2015  2016-2023 | 30.6%  29.9%  39.5% | 21.0%  29.6%  49.4% | 23.4%  27.1%  49.5% | 18.7%  31.3%  50.0% | <0.001 |
| Annual HTx center volume  Low (<15)  Medium (15-35)  High (>35) | 23.5%  51.5%  25.0% | 25.9%  50.6%  23.5% | 26.7%  46.0%  27.4% | 22.6%  40.6%  36.8% | <0.001 |

Abbreviations: BMI (body mass index), ECMO (extracorporeal membrane oxygenation), HCM (hypertrophic cardiomyopathy), HTx (heart transplant), IABP (intra-aortic balloon pump), ICM (ischemic cardiomyopathy), NIDCM (nonischemic dilated cardiomyopathy), RCM (restrictive cardiomyopathy), TAH (total artificial heart), VAD (ventricular assist device), WL (waitlist).

*At listing

^1^Allocation status of 2 (1999-2018) and 5 and 6 (2018-Present)

^2^Allocation status of 1B (1999-2018) and 4 (2018-Present)

^3^Allocation status of 1A (1999-2018) and 1, 2, and 3 (2018-Present)

**Supplementary Table S2:** Donor and donor-recipient matching data

|  | White  (n=33,977) | Black  (n=10,345) | Hispanic  (n=4,331) | Asian  (n=1,590) | *p-value* |
| --- | --- | --- | --- | --- | --- |
| Age (years) | 31 (22-41) | 31 (23-40) | 30 (22-40) | 30 (22-40) | <0.001 |
| Sex (male) | 76.6% | 66.8% | 74.5% | 76.9% | <0.001 |
| Weight (kg) | 83.1±19.0 | 84.2±19.6 | 79.4±18.3 | 75.6±17.7 | <0.001 |
| BMI (kg/m^2^) | 27.3±5.9 | 27.6±6.1 | 26.8±5.8 | 26.0±5.8 | <0.001 |
| Race and ethnicity  White  Black  Hispanic  Asian | 68.6%  14.4%  14.5%  1.5% | 62.5%  19.7%  15.4%  1.5% | 53.2%  12.5%  30.8%  2.2% | 54.6%  15.1%  24.4%  4.2% | <0.001 |
| History of hypertension | 14.7% | 14.9% | 12.6% | 12.8% | <0.001 |
| History of diabetes | 3.3% | 3.1% | 3.8% | 3.0% | 0.102 |
| History of cigarette use (>20PY) | 17.9% | 15.6% | 13.9% | 11.5% | <0.001 |
| Cause of death  Anoxia  Head trauma  CVA | 26.7%  51.2%  19.4% | 29.9%  49.1%  18.2% | 28.4%  49.7%  19.5% | 29.3%  48.1%  19.9% | <0.001 |
| Ischemic time (hours) | 3.29±1.11 | 3.28±1.14 | 3.27±1.14 | 3.34±1.10 | 0.171 |
| PHM ratio | 1.03±0.18 | 1.04±0.18 | 1.05±0.19 | 1.09±0.20 | <0.001 |
| HLA mismatch  0-3  4-6 | 16.2%  83.8% | 9.5%  90.5% | 13.6%  86.4% | 11.4%  88.6% | <0.001 |
| HLA-A mismatch  0-1  2 | 51.6%  48.4% | 41.0%  59.0% | 48.6%  51.4% | 46.1%  53.9% | <0.001 |
| HLA-B mismatch  0-1  2 | 30.2%  69.8% | 23.3%  76.7% | 28.1%  71.9% | 22.5%  77.5% | <0.001 |
| HLA-DR mismatch  0-1  2 | 46.0%  54.0% | 42.0%  58.0% | 43.6%  56.4% | 40.2%  59.8% | <0.001 |

Abbreviations: BMI (body mass index), CVA (cerebrovascular accident), HLA (human leukocyte antigen), PHM (predicted heart mass), PY (pack-year).

.

**Supplementary Table S3:** Transplant peri- and postoperative data

|  | White  (n=33,977) | Black  (n=10,345) | Hispanic  (n=4,331) | Asian  (n=1,590) | *p-value* |
| --- | --- | --- | --- | --- | --- |
| Medical condition at Tx  Not hospitalized  Hospitalized, not in ICU  ICU | 49.3%  16.3%  34.4% | 44.8%  16.7%  38.6% | 43.1%  15.5%  41.4% | 42.2%  13.6%  44.2% | <0.001 |
| Induction agent  IL-2 receptor antagonist^1^  Polyclonal ALG/ATG^2^  Steroids | 25.7%  19.5%  63.6% | 29.9%  19.2%  66.9% | 23.9%  19.8%  67.4% | 21.4%  23.8%  64.1% | <0.001  <0.001 <0.001 |
| LOS (days) | 15 (10-23) | 16 (11-24) | 15 (11-22) | 15 (11-23) | <0.001 |
| Events prior to discharge  Dialysis  PPM  Stroke  Acute rejection | 11.3%  3.0%  3.0%  17.5% | 11.9%  2.5%  2.8%  20.8% | 10.9%  2.0%  2.7%  17.4% | 9.3%  2.7%  3.5%  12.8% | 0.016  <0.001  0.194  <0.001 |
| Treated for acute rejection episodes within 1yr | 22.9% | 25.0% | 22.0% | 16.2% | <0.001 |
| Cause of death  Cardiovascular  Infection  Malignancy  Graft failure  MOF  Other | 13.4%  15.2%  12.9%  8.8%  7.9%  41.9% | 23.5%  14.1%  4.8%  14.5%  6.7%  36.5% | 17.9%  18.8%  5.5%  11.1%  8.7%  38.0% | 17.3%  15.8%  7.0%  10.1%  8.9%  41.0% | <0.001 |

Abbreviations: ALG (anti-lymphocyte globulin), ATG (anti-thymocyte globulin), ICU (intensive care unit), IL-2 (interleukin 2), LOS (length of stay), MOF (multiple organ failure), PPM (permanent pacemaker), Tx (transplant).

^1^Basiliximab, Daclizumab

^2^ATGAM, Thymoglobin

**Supplementary Table S4:** Recipient and donor information between heart allocation policy eras

|  | Pre-HAP Era  (Nov. 1^st^, 2013 – Oct. 17^th^, 2018) | | | Post-HAP Era  (Oct. 18^th^, 2018 – Sept. 30^th^, 2023) | | |
| --- | --- | --- | --- | --- | --- | --- |
|  | White  (n=7,533) | Black  (n=2,620) | *p-value* | White  (n=7,864) | Black  (n=3,145) | *p-value* |
| Recipient data | | | | | | |
| Age (years) | 58 (49-64) | 54 (44-61) | <0.001 | 58 (49-65) | 54 (42-61) | <0.001 |
| Sex (male) | 75.3% | 67.3% | <0.001 | 73.9% | 67.6% | <0.001 |
| Weight (kg) | 84.9±17.7 | 85.8±18.7 | 0.028 | 85.0±17.6 | 86.4±19.1 | <0.001 |
| BMI (kg/m^2^) | 27.7±4.7 | 28.0±5.0 | 0.013 | 27.8±4.8 | 28.2±5.3 | <0.001 |
| Primary etiology  NIDCM  ICM  HCM  RCM | 49.8%  36.5%  3.6%  3.5% | 74.5%  16.6%  1.2%  4.1% | <0.001 | 49.8%  32.7%  4.4%  4.5% | 76.1%  13.7%  1.7%  5.0% | <0.001 |
| History of diabetes | 27.0% | 28.6% | 0.099 | 26.8% | 30.0% | <0.001 |
| History of cigarette use | 48.6% | 42.2% | <0.001 | 43.4% | 37.9% | <0.001 |
| Highest educational level  High school or below  College or above | 37.5%  62.5% | 45.6%  54.4% | <0.001 | 35.9%  64.2% | 46.0%  54.1% | <0.001 |
| Insurance type  Medicaid or donation  Medicare or other govt. sponsored  Private or self-funded | 23.2%  21.5%  55.2% | 32.9%  26.0%  41.1% | <0.001 | 27.3%  19.4%  53.2% | 38.6%  19.7%  41.7% | <0.001 |
| Days on WL | 111 (31-306) | 120 (35-353) | <0.001 | 24 (8-95) | 24 (9-87) | 0.165 |
| Acuity status at transplant  Low^1^  Medium^2^  High^3^ | 3.3%  30.0%  66.7% | 1.8%  28.9%  69.3% | <0.001 | 6.6%  18.5%  75.0% | 3.0%  15.2%  81.8% | <0.001 |
| Donor data | | | | | | |
| Age (years) | 31 (23-40) | 31 (23-39) | 0.385 | 32 (25-40) | 32 (25-40) | 0.018 |
| Sex (male) | 70.5% | 70.1% | 0.690 | 72.7% | 73.2% | 0.615 |
| Weight (kg) | 84.1±19.6 | 84.9±19.9 | 0.045 | 85.0±19.9 | 86.1±20.1 | 0.006 |
| BMI (kg/m^2^) | 27.7±6.1 | 28.0±6.2 | 0.041 | 28.0±6.3 | 28.2±6.4 | 0.069 |
| Race  White  Black  Hispanic  Asian | 67.6%  15.2%  14.6%  1.5% | 59.9%  21.0%  16.2%  1.8% | <0.001 | 65.5%  15.6%  16.0%  1.4% | 60.7%  20.1%  16.7%  1.6% | <0.001 |
| History of hypertension | 16.6% | 16.6% | 0.967 | 16.1% | 14.7% | 0.065 |
| History of diabetes | 4.2% | 3.3% | 0.040 | 3.9% | 3.2% | 0.065 |
| History of cigarette use (>20PY) | 12.2% | 10.5% | 0.019 | 12.9% | 12.8% | 0.825 |
| Cause of death  Anoxia  Head trauma  CVA | 36.0%  45.3%  16.1% | 35.6%  45.7%  15.9% | 0.937 | 47.7%  37.8%  11.9% | 45.9%  39.9%  11.6% | 0.221 |
| Ischemic time (hours) | 3.1±1.0 | 3.1±1.1 | 0.547 | 3.6±1.3 | 3.6±1.2 | 0.912 |
| D/R data | | | | | | |
| PHM ratio | 1.02±0.17 | 1.03±0.17 | 0.011 | 1.03±0.17 | 1.04±0.17 | 0.003 |
| HLA mismatch  0-3  4-6 | 15.8%  84.3% | 9.8%  90.2% | <0.001 | 16.4%  83.6% | 9.2%  90.8% | <0.001 |
| Recipient cause of death | | | | | | |
| 30-days  Cardiovascular  Infection  Malignancy  Graft failure  MOF | 15.4%  9.2%  0.0%  22.8%  18.8% | 20.3%  7.8%  0.0%  26.6%  15.6% | 0.792 | 14.4%  13.1%  0.5%  14.0%  14.4% | 13.0%  11.6%  0.0%  21.7%  10.1% | 0.648 |
| 1-year  Cardiovascular  Infection  Malignancy  Graft failure  MOF | 12.6%  22.8%  2.0%  13.7%  17.1% | 15.0%  19.1%  1.0%  17.5%  12.9% | 0.312 | 11.8%  27.3%  1.4%  9.0%  12.0% | 15.1%  24.4%  0.4%  11.6%  11.6% | 0.463 |
| Total  Cardiovascular  Infection  Malignancy  Graft failure  MOF | 14.9%  20.5%  9.8%  9.7%  10.0% | 21.6%  15.5%  4.6%  14.1%  7.6% | <0.001 | 13.2%  25.6%  4.1%  7.9%  10.1% | 23.5%  20.2%  1.5%  11.9%  8.1% | <0.001 |

Abbreviations: BMI (body mass index), ECMO (extracorporeal membrane oxygenation), HCM (hypertrophic cardiomyopathy), IABP (intra-aortic balloon pump), ICM (ischemic cardiomyopathy), NIDCM (nonischemic dilated cardiomyopathy), RCM (restrictive cardiomyopathy), TAH (total artificial heart), VAD (ventricular assist device), WL (waitlist).

*At listing

^1^Allocation status of 2 (1999-2018) and 5 and 6 (2018-Present)

^2^Allocation status of 1B (1999-2018) and 4 (2018-Present)

^3^Allocation status of 1A (1999-2018) and 1, 2, and 3 (2018-Present)
